# Possibility of chest wall dose reduction using volumetric-modulated arc therapy (VMAT) in radiation-induced rib fracture cases: comparison with stereotactic body radiation therapy (SBRT)

**DOI:** 10.1093/jrr/rry012

**Published:** 2018-03-22

**Authors:** Yu Murakami, Masahiro Nakano, Masahiro Yoshida, Hideaki Hirashima, Fumiya Nakamura, Junichi Fukunaga, Taka-aki Hirose, Yasuo Yoshioka, Masahiko Oguchi, Hideki Hirata

**Affiliations:** 1 Division of Medical Quantum Sciences, Department of Health Sciences, Graduate School of Medical Sciences, Kyushu University, 3-1-1, Maidashi, Higashi-ku, Fukuoka 812-8582, Japan; 2 Radiation Oncology Department, Cancer Institute Hospital, Japanese Foundation for Cancer Research, 3-8-31 Ariake, Koto-ku, Tokyo 135-8550, Japan; 3 Department of Radiation Oncology and Image-applied Therapy, Graduate School of Medicine, Kyoto University, 54 Shogoin-Kawaharacho, Sakyo-ku, Kyoto 606-8507, Japan; 4 Division of Radiology, Department of Medical Technology, Kyushu University Hospital, 3-1-1, Maidashi, Higashi-ku, Fukuoka 812-8582, Japan

**Keywords:** stereotactic body radiation therapy (SBRT), three-dimensional conformal radiation therapy (3D-CRT), volumetric-modulated arc therapy (VMAT), non–small cell lung cancer (NSCLC), chest wall pain, radiation-induced rib fracture (RIRF)

## Abstract

The present study compares dosimetric parameters between volumetric-modulated arc therapy (VMAT) and 3D conformal radiation therapy (3D-CRT) in lung tumors adjacent to the chest wall treated with stereotactic body radiation therapy (SBRT). The study focused on the radiation dose to the chest wall of 16 patients who had developed radiation-induced rib fractures (RIRF) after SBRT using 3D-CRT. The targets in all patients were partially overlapping with the fractured ribs, and the median overlapping rib–PTV distance was 0.4 cm. Stereotactic body radiation therapy was re-planned for all patients. The prescribed dose was 48 Gy in four fractions to cover at least 95% of the planning target volume (PTV). Evaluated dosimetric factors included D_98%_ and the conformation number (CN) of the PTV, the D_2cm_^_3_^, V_40_ and V_30_ of the fractured ribs, the V_30_ of the chest wall, and the D_mean_, V_20_ and V_5_ of the lung. A comparison of 3D-CRT with the VMAT plan for PTV revealed that CN was significantly improved in the VMAT plan, whereas D_98%_ did not significantly differ between the two plans. Regarding organs at risk (OARs), the D_2cm_^_3_^, V_40_ and V_30_ of fractured ribs, the V_30_ of the chest wall, and the D_mean_, V_20_ and V_5_ of the lung, were significantly decreased in the VMAT plan. We concluded that the dose to OARs such as ribs and chest wall could be reduced with improved target conformity using VMAT instead of 3D-CRT for SBRT to treat peripheral lung tumors.

## INTRODUCTION

Stereotactic body radiation therapy (SBRT) allows escalation of the fractional dose to the target while minimizing the radiation dose to normal tissue, resulting in excellent local control and survival rates, with limited toxicity in patients with medically inoperable early-stage non–small cell lung cancer (NSCLC) [1].

Three-dimensional conformal radiation therapy (3D-CRT), dynamic conformal arc therapy (DCAT) and volumetric-modulated arc therapy (VMAT) are three main irradiation techniques in lung SBRT [2, 3]. The 3D-CRT, which is the conventional irradiation technique for SBRT, uses multiple static fields (from 7 to 11 fields in most cases) including coplanar and noncoplanar beams. Though this technique has a highly conformal dose distribution by concentrating the dose to the target from all directions, a steep dose gradient between the target and organs at risk (OARs) adjacent to the target is difficult to achieve. For example, the chest wall is irradiated with a high dose during treatment for peripheral lung tumors adjacent to chest wall. Therefore, adverse events such as radiation-induced rib fracture (RIRF) and chest wall pain occur in many cases after SBRT [4–8]. Several studies have reported that the incidence of RIRF after SBRT for lung tumors was 23–37% [9–12]. Clinical factors such as target location [9], female sex [10, 13] and rib–tumor distance [12–14] are known to increase the risk of RIRF and chest wall pain after SBRT for lung tumors. Regarding dosimetric factors, a high-dose-irradiated volume of the chest wall is thought to contribute to the increased the incidence of such adverse events [7, 8, 15]. VMAT, a novel irradiation technique for SBRT, should reduce the dose to the chest wall through inverse planning. Ding *et al.* compared dosimetric parameters of OARs in lung cancer adjacent to the chest wall (average 2 cm) between a static field plan and VMAT. They found that the VMAT plan significantly decreased the V_30_ for the rib and chest wall, and the V_20_ for the lung, except in one patient [16]. These findings suggest that the VMAT plan reduces the OARs dose compared with the static field plan. However, that study did not take differences in the target dose into account when comparing the two plans. Furthermore, whether or not the dose to the chest wall can decrease with VMAT is unknown in cases where the target is overlapping with the fractured ribs in patients who develop RIRF. If VMAT could reduce the dose to the chest wall in these patients, then the risk of such adverse events after SBRT should be decreased.

The present study aimed to determine whether or not the dose to the chest wall could be decreased by VMAT in patients with RIRF. The study also compared the dose to the target and to the OARs adjacent to the target between the two plans.

## MATERIALS AND METHODS

### Patient characteristics

The patients who developed RIRF after SBRT at Kyushu University between April 2003 and May 2007 were retrospectively reviewed. The analysis included 28 ribs in 16 patients in this study. The inclusion criteria for this study were as follows: (i) early-stage primary or metastatic lung cancer; (ii) prescribed 48 Gy in four fractions to the isocenter; (iii) developed RIRF after SBRT with 3D-CRT and (iv) planning target volume (PTV) partially overlapping with the fractured rib, and <0.6 cm overlap between the rib and the PTV. Patients with an overlapping rib–PTV distance >0.6 cm were excluded from the study due to the difficulty in reducing the rib dose. The indications for SBRT at Kyushu University have been described in detail by Asai *et al.* [12]. Table 1 summarizes more information about the patients, and Table 2 shows details of the fractured ribs. We defined overlapping rib–PTV distance as the overlapped maximum distance between the lateral border of the PTV and the rib surface on three orthogonal planes. The median overlapping rib–PTV distance was 0.4 cm. The maximum number of RIRFs was three in one patient. We selected patients who developed RIRF after SBRT with 3D-CRT as being good candidates in which to reduce radiation doses to the chest wall. The Ethics Committee at Kyushu University approved the study.

**Table 1.** Patient characteristics

Characteristic	No. (median)
Number of patients	16
Sex	
Males	5
Females	11
Age	54–92 (74)
Diagnosis	
Primary	14
Metastasis	2
Tumor size [cm]	1.2–3.8 (2.4)
PTV volume [cm^3^]	10.28–59.87 (36.51)

**Table 2.** Details of the fractured ribs

Characteristics	No. (median)
Number of fractured ribs	28
Location of fractured ribs	
Right	12
Costae verae (rt1/rt2/rt3/rt4/rt5/rt6/rt7)	12 (–/1/3/3/2/1/2)
Costae spuriae (rt8/rt9/rt10/rt11/rt12)	
Left	16
Costae verae (lt1/lt2/lt3/lt4/lt5/lt6/lt7)	13 (–/–/3/3/3/2/2)
Costae spuriae (lt8/lt9/lt10/lt11/lt12)	3 (2/1/–/–/–)
Overlapping rib–PTV distance [cm]	0.2–0.6 (0.4)

ltx = developed RIRF with X-place in left ribs, rtx = developed RIRF with X-place in right ribs.

### Target and risk organs delineation

**Fig. 1.**
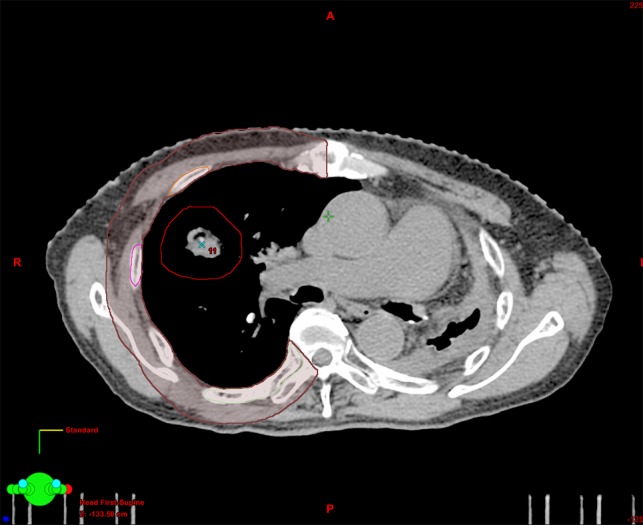
The outline indicates how to contour the chest wall.

The gross tumor volume (GTV) was contoured on each axial CT slice using a pulmonary window setting. The clinical target volume (CTV) was equivalent to the GTV. The internal target volume (ITV) was defined based on 3D tumor motion using fluoroscopy. The PTV was created from the ITV by adding a uniform margin of 5 mm in all directions. All ribs were contoured just on the rim (except for cartilage) under the bone window setting. The chest wall was contoured essentially as described in Refs. [15] and [17] by uniformly expanding the lung by 2 cm, including the exit site of the spinal nerve root and part of the sternum, and excluding the spinal cord and soft tissue in mediastinum in terms of the ipsilateral lung (Fig. 1).

### Treatment planning

All patients were retrospectively re-planned for 3D-CRT in accordance with the JCOG0702 protocol [18], and VMAT plans were newly created to be compared with the 3D-CRT plans. The prescription of original 3D-CRT plans was defined at the isocenter, and the plans were calculated with a pencil beam convolution (PBC) algorithm. The prescription of the original plans was changed from the point prescription to the volume prescription to equalize the target dose distribution of 3D-CRT and VMAT plans. Additionally, Acuros XB (AXB) was adopted instead of the PBC algorithm because it is known to have high accuracy for the dose calculation in lung SBRT plans with small PTVs compared with PBC algorithms [19]. The treatment planning system (TPS) was Eclipse, ver. 11 (Varian Medical Systems, Palo Alto, CA, USA), and the treatment beam was 6-MV or 10-MV photon beams using a TrueBeam STx linear accelerator (Varian Medical Systems). The prescribed dose was 48 Gy in four fractions, and the planning objectives aimed to cover the PTV with 95% of the prescribed dose (D_95%_ to 48 Gy) in both plans. The homogeneity index (HI) was defined as a ratio of the maximum dose to the minimum dose in the PTV. An HI of <1.6 was adopted in this study [18]. The dose calculation was performed with a calculation grid resolution of 2.5 mm, and a heterogeneity correction was used in all the plans.

A radiation oncologist at Kyushu University slightly modified some field arrangements (within ± 10°) in all 3D-CRT plans in order to avoid a deformation of the dose distribution due to the change in calculation algorithm and prescription, while generally keeping the field arrangements of the original plan and considering target conformity. The field arrangement for the 3D-CRT plan was 7 or 8 using a non-coplanar technique. A margin of 5 mm was applied from the PTV to the multileaf collimator (MLC) leaves for all fields.

The field arrangement for the VMAT plan included two partial arcs of 200° (e.g. 339° to 179° for left lung, and 21° to 181° for right lung) with a coplanar technique in terms of the ipsilateral lung to maximally reduce doses to the contralateral lung. All plans used 30° collimator rotation for two different arcs. The dose constraints for all lungs were V_20_ ≤ 5% (<5% of the volume receiving 20 Gy), V_10_ ≤ 10%, V_5_ ≤ 15% and D_mean_ ≤ 4 Gy, and the doses for the fractured ribs and chest wall were maximally reduced. The irradiated dose to the intact ribs was also maximally reduced.

### Dosimetric parameters

The D_98%_ (dose received by ≥98% of the PTV volume) and conformation number (CN) of the PTV were calculated. Riet *et al.* have defined the *CN* as

(1) $$\mathrm{CN}=\frac{{\mathrm{Vt}}_{100}^{2}}{({\mathrm{Vt}}_{\mathrm{vol}}\;\cdot \;V_{100})},$$

where *Vt*_100_ is the target volume receiving at least the prescribed dose, *Vt*_vol_ is the target volume, and *V*_100_ is the total volume receiving at least the prescribed dose [20]. A value close to unity means identical target coverage.

The D_2cm_^_3_^ (dose received by 2 cm^3^ of the objective structure), the V_40_ and V_30_ of the fractured ribs, the V_30_ of the chest wall, and the D_mean_, V_20_ and V_5_ of the lungs were also calculated.

### Statistical analysis

Data were statistically analyzed using JMP Pro 11 software (SAS Institute, Inc., Cary, NC, USA). All *P* values were two-sided, with a level of significance set at 5%. The dosimetric parameters of the PTV and OARs were compared between the 3D-CRT and VMAT plans using the Wilcoxon signed rank test.

## RESULTS

**Fig. 2.**
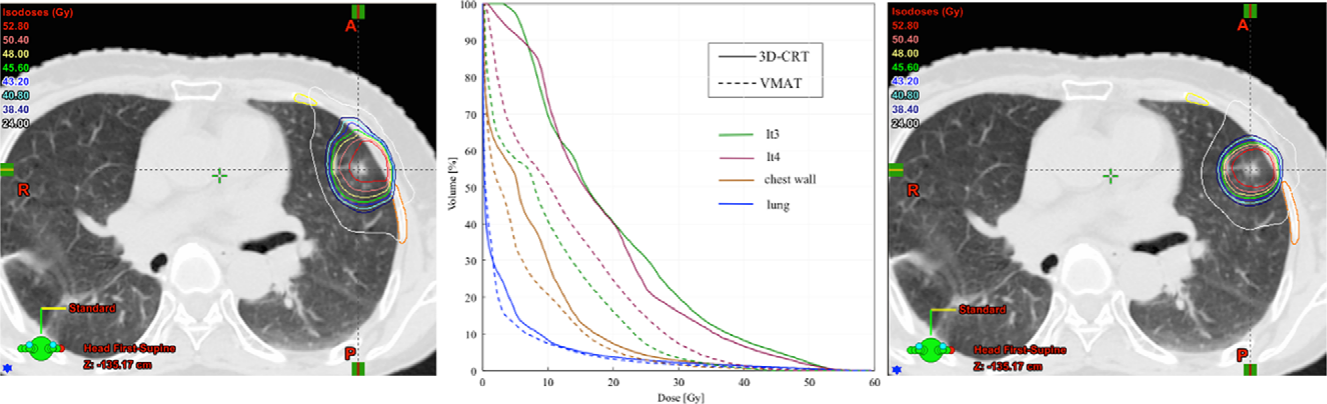
The dose distributions and dose–volume histograms (DVHs) of normal tissues showing 3D-CRT and VMAT plans for one patient with primary cancer in left lung. Dose distribution with 3D-CRT plan (left); dose distribution with VMAT plan (right). Values of CN in 3D-CRT and VMAT plans were 0.57 and 0.79, respectively. ltx = developed RIRF with X-place in left ribs.

A summary of the dosimetric parameters is shown in Table 3. Regarding the target, D_98%_ did not significantly differ between the two plans, whereas CN was significantly improved in the VMAT plans (*P* < 0.0001). Regarding the OARs, all dosimetric parameters of the fractured ribs were significantly decreased in the VMAT, compared with in the 3D-CRT plans (*P* < 0.0001). The V_30_ of the chest wall and all dosimetric parameters of the lung were significantly decreased in the VMAT plans (*P* < 0.0001 for both, except for V_5_ of lung, *P* = 0.0002). Especially, irradiated volumes of fractured ribs were remarkably decreased in the VMAT plans, and the reduction rate of the V_40_ and V_30_ for fractured ribs were −52.4% and −41.5%, respectively. Figure 2 shows the dose distribution and dose–volume histogram (DVH) for one patient in whom the CN value improved in the VMAT, compared with in the 3D-CRT plan (0.79 vs 0.57). The dose distribution was more conformable using the VMAT, than the 3D-CRT plan. The irradiated volume to the fractured ribs (lt3, lt4) and the chest wall was decreased in the VMAT plan. The irradiated lung volume also decreased, whereas the lung volume receiving <2 Gy increased in the VMAT plan. These results revealed that the irradiated volume of the OARs adjacent to the target was reduced by the VMAT, compared with the 3D-CRT plans, although the low-dose irradiated lung volume increased.

**Table 3.** Summary of dosimetric parameters

Objects	Criteria	3D-CRT	VMAT	^a^Ratio [%]	*P* value
PTV	D_98%_ [Gy]	47.00 (46.88–47.13)	46.85 (46.56–47.08)	−0.3	0.07
	CN	0.58 (0.53–0.62)	0.83 (0.79–0.87)	43.1	<0.0001
Rib	D_2 cm_^_3_^ [Gy]	40.46 (31.36–47.97)	26.95 (21.44–36.67)	−33.4	<0.0001
	V_40_ [cm^3^]	2.12 (1.05–2.93)	1.01 (0.08–1.70)	−52.4	<0.0001
	V_30_ [cm^3^]	2.87 (2.20–4.25)	1.68 (0.78–2.91)	−41.5	<0.0001
Chest wall	V_30_ [cm^3^]	29.97 (22.20–52.35)	19.65 (10.17–33.69)	−34.4	<0.0001
Lung	D_mean_ [Gy]	4.80 (3.28–6.18)	4.13 (2.83–5.26)	−14.0	<0.0001
	V_20_ [%]	6.63 (3.82–9.29)	5.46 (3.13–8.20)	−17.7	<0.0001
	V_5_ [%]	21.33 (16.36–28.88)	18.18 (12.87–21.26)	−14.8	0.0002

Value = median (interquartile range), D_98%_ = dose received by at least 98% of volume of the PTV, CN = conformation number, D_2cm_^_3_^ = dose received by volume of 2 cm^3^ of the fractured rib, V_x_ = volume received by at least *x* Gy of the dose, ^a^Ratio [%] = ratio of the median value between VMAT and 3D-CRT (VMAT/3D-CRT).

## DISCUSSION

This study investigated that whether or not the dose to the chest wall could be decreased by VMAT in patients with RIRFs and compared the dose to the target and the OARs adjacent to the target between the two plans. The present results indicate that VMAT reduced the dose to the chest wall, ribs and lung, while offering more conformable dose distribution for the target compared with 3D-CRT in SBRT for a peripheral lung tumor adjacent to the chest wall.

In lung cancer patients, Ding *et al.* compared the dosimetric parameters of OARs adjacent to the chest wall (average 2 cm) between a static field plan and VMAT, and reported that VMAT significantly reduced the dose to chest wall and ribs, and the average improvement rates of the V_30_ were 74.3% and 60.8%, respectively [16]. However, these findings did not take differences in the target dose into account between the two plans. Indeed, a highly conformal dose distribution for the target with a steep dose gradient neighboring some healthy tissues causes underdosing of the target in VMAT plans. The present study compared the target and OARs doses between VMAT and 3D-CRT plans in RIRF patients (overlapping rib–PTV distance >0.2 cm). The VMAT plans achieved better OARs sparing without compromising target dose coverage. The median reduction rates of the V_30_ of the chest wall and fractured ribs were 34.4% and 41.5%, respectively (Table 3). It is suggested that VMAT could decrease the dose to the OARs adjacent to the target, such as chest wall and ribs, without underdosing of the target, even when the target is partially overlapping with these OARs. Ding *et al.* also reported that one patient observed an increase in the V_20_ of the ipsilateral lung with the VMAT plan. However, this tendency was not shown in this study. We considered that this was due to differences in the dose constraints and the evaluated volume of the lung. We noted that the low-dose irradiated lung volume (i.e. volume of lung irradiated with <5 Gy) might increase, since the V_5_ increased by 2.3% in one of 16 patients (2.3%) in this study.

Some investigators have described risk factors of RIRF and chest wall pain. Asai *et al.* analyzed the risk factors for RIRF using receiver operating characteristics (ROC) curves and found a close correlation between D_max_, V_40_ and V_30_ for the ribs and RIRFs after SBRT [12]. They estimated the risk of RIRFs in 3 years was 51.6% when the V_40_ of the ribs above 0.29 cm^3^, whereas 2.01% when the V_40_ was less 0.29 cm^3^. Regarding the V_30_ of the ribs, estimated the risk was 45.8% when the V_30_ was above 1.35 cm^3^, while 2.2% when the V_30_ was less 1.35 cm^3^. Comparison of our results with these cut-off values, V_40_ ≤ 0.29 cm^3^ occurred in 1 of a total of 28 ribs (3.6%) with 3D-CRT plans and in 10 ribs (35.7%) with VMAT plans, and V_30_ ≤ 1.35 cm^3^ occurred in 4 ribs (14.3%) with 3D-CRT plans and in 10 ribs (35.7%) with VMAT plans, respectively. Eight ribs met the both cut-off values (i.e. V_40_ ≤ 0.29 cm^3^ and V_30_ ≤ 1.35 cm^3^) in “VMAT” plans. It is suggested that 8 of total the 28 ribs (28.6%) could be have a decreased risk of RIRF (down to 2%) by using VMAT instead of 3D-CRT in SBRT. Welsh *et al.* assessed the correlations between the chest wall dose and adverse events, and found that the V_30_ for the chest wall closely correlates with the incidence of skin toxicity and chest wall pain after SBRT [7]. They estimated that the risk of skin reaction rate was 44% and 22% when V_30_ of the chest wall was above or less than 50 ml, respectively. The risk of chest wall pain was estimated at 18% and 2.7% when the V_30_ was above or less than 30 ml, respectively. When comparing our results with these values, the V_30_ of the chest wall ≤ 50 ml was 12 of 16 patients (75%) with 3D-CRT plans, whereas 16 patients (100%) with VMAT plans showed the V_30_ ≤ 50 ml in terms of estimated the risk of skin toxicity. About the risk of chest wall pain, the V_30_ of the chest wall ≤ 30 ml was 8 of 16 patients (50%) with 3D-CRT plans, while 10 of 16 patients (62.5%) with VMAT plans showed V_30_ ≤ 30 ml. It is suggested that VMAT plans could reduce the dose to the ribs and chest wall compared with 3D-CRT plans, and that this might help decrease the risk of adverse events such as RIRF and chest wall pain.

Some studies reported that a short distance from the rib and chest wall to the tumor (<1.0–2.0 cm) increases the of risk of RIRFs [12–14]. However, in the evaluation of risk of RIRF, the use of rib–tumor distance as a risk factor is not appropriate because the dose to the ribs and chest wall can be decreased by use of intensity-modulated radiation therapy (IMRT) techniques, including VMAT. In our results, we found that VMAT reduced these OARs doses; the median reduction rates of V_40_ and V_30_ for fractured ribs and V_30_ for chest wall were −52.4%, −41.5% and −34.4%, respectively. Therefore, when IMRT techniques, including VMAT, are used to treat a peripheral lung tumor adjacent to the chest wall, evaluation of rib–tumor distance as a risk factor may not be appropriate.

One concern about lung SBRT using VMAT is the interplay effect. The interplay effect causes overdosage and underdosage within the target due to interplay between respiration-induced tumor motion and MLC motion, and single-arc and single-fraction treatment correlated with noticeable dose deviations [21, 22]. According to these findings, lung SBRT with VMAT might cause dose deviations in the target due to the interplay effect, because this treatment is generally completed in a few fractions with 1 or 2 arcs per fraction. Ong *et al.* compared convolved static dose measurements, including 10 different respiratory phases, with dynamic dose measurement in VMAT plans by gamma analysis with evaluation criteria of 3% dose difference and 1 mm distance-to-agreement (DD/DTA: 3%/1 mm), and reported that the average surface with gamma >1 was 1.3% in 1 arc, and further decreased to 0.3% in 2 arcs [22]. The dose deviations in the center of the PTV averaged 1–2%. Therefore, it is suggested that the dosimetric effects of interplay might be negligible in lung SBRT with VMAT, especially the one consists of 2 arcs.

Our study has several limitations. We did not include patients with an overlapping rib–PTV distance of >0.6 cm. However, our selected cases included patients in whom RIRFs occurred at various locations in lung (Table 2). Our results offer a benchmark for considering the risk of RIRF and chest wall pain in clinical practice. Finally, we did not apply clinical approaches, such as adjustments to the MLC position on the side of the chest wall, in the re-planned 3D-CRT. The 3D-CRT plans were created based on the JCOG0702 protocol [18], that is, 5 mm of MLC margin was adopted in the plans. The authors would like to note that the results of this study should not be interpreted as saying that a VMAT plan is generally superior to a 3D-CRT plan, because 3D-CRT still retains the capacity for reduced OAR doses by adjusting an edge of the MLC in a more sophisticated way than is described by the JCOG0702 protocol. If the MLC margin was to be diminished (that is, from 5 to 0 mm) on the side of the chest wall, the dose to the chest wall could be reduced. However, VMAT can reduce the dose to the chest wall and ribs, even when the target is partially overlapping with these OARs. We aim to clarify the relationships between chest wall dose and occurrence of RIRF in lung SBRT with VMAT in the future.

In conclusion, the VMAT plan allowed a reduction in the radiation dose to the chest wall, ribs and lung, while improving target conformity compared with 3D-CRT for SBRT used to treat peripheral lung tumors. Reducing the dose to the OARs helps to decrease the risk of some adverse events, such as RIRF and chest wall pain, after SBRT.
